# The Trophic Cascade Effects of Marine Mesozooplankton: Theory, Dynamics, and Responses to Global Change

**DOI:** 10.3390/microorganisms14030697

**Published:** 2026-03-19

**Authors:** Mianrun Chen

**Affiliations:** 1 Nansha Islands Coral Reef Ecosystem National Observation and Research Station/South China Sea Development Research Institute, Ministry of Natural Resources (Remote Sensing Technology Application Center of South China Sea, MNR), Guangzhou 510300, China; cmrandy@foxmail.com; 2 Technology Innovation Center for South China Sea Remote Sensing, Surveying and Mapping Collaborative Application, Guangzhou 510300, China; 3 Southern Marine Science and Engineering Guangdong Laboratory (Zhuhai), Zhuhai 519082, China

**Keywords:** mesozooplankton grazing, trophic cascades, selective feeding, climate change drivers, biological pump, planktonic food web

## Abstract

Marine mesozooplankton (0.2–20 mm), as a critical trophic link between primary producers and higher trophic levels, are pivotal drivers of trophic cascades regulating pelagic ecosystem structure and function. This review synthesizes recent advances in understanding mesozooplankton-mediated trophic cascades (MMTC), with a focus on selective feeding mechanisms, and presents an original, integrated quantitative framework that fills gaps in quantification and prediction of MMTC. This framework includes the following: a dual-pathway conceptual model distinguishing density-mediated and trait-mediated cascades; a three-level grazing rate correction model addressing long-standing underestimations of mesozooplankton direct grazing rate on phytoplankton; a comprehensive Cascade Strength Index for quantifying cascade intensity; an extended numerical model—NPMZ model (Nutrient–Phytoplankton–Microzooplankton–Mesozooplankton) for simulating MMTC dynamics and their biogeochemical impacts. The review further elucidates the spatiotemporal heterogeneity of MMTC and its implications for plankton community size structure and biological carbon pump efficiency. It also systematically assess the combined impacts of global change drivers (ocean warming, acidification, eutrophication) on MMTC and their ecological consequences. This review advances the theoretical framework of marine trophic cascade research by establishing a unified quantitative paradigm for MMTC and provides mechanistic insights and predictive tools for understanding how climate change modulates pelagic food web dynamics and marine ecosystem services. Moreover, the proposed integrated research paradigm combining molecular tools, multi-factor experiments, and high-resolution numerical modeling offers a critical roadmap for future MMTC research in the Anthropocene. This provides a scientific basis for the conservation and adaptive management of marine ecosystems under global change.

## 1. Introduction

Trophic cascades, defined as the propagation of indirect effects across multiple trophic levels initiated by perturbations in predator abundance, constitute a cornerstone of ecological theory with profound implications for carbon cycling and ecosystem stability [1]. These intricate dynamics describe how predators indirectly influence lower trophic levels, often leading to ecosystem-wide changes [2,3]. These cascades are fundamental in marine ecosystems by shaping food web structures, influencing habitat integrity, and contributing to ecosystem regime shifts [4,5,6].

Marine mesozooplankton (0.2–20 mm), predominantly comprising copepods, cladocerans, and gelatinous taxa, are central actors in these cascades. Occupying a critical trophic junction, they serve as both consumers of phytoplankton and predators of microzooplankton (e.g., ciliates, heterotrophic dinoflagellates), thereby exerting direct and indirect control over primary production [7]. Historically conceptualized as simplified linear chains, marine food webs are now understood as highly interconnected networks characterized by omnivory and mixotrophy, where selective feeding—driven by prey size, motility, nutritional value, and chemical cue—is the rule rather than the exception [8]. This selectivity transforms mesozooplankton into active agents that shape plankton community composition. When mesozooplankton preferentially graze on microzooplankton, they indirectly release phytoplankton from grazing pressure, initiating a classic trophic cascade that can enhance primary production and alter phytoplankton size spectra [3,9]. Understanding their role in mediating trophic cascades is essential, especially given the pervasive influence of global change stressors on marine environments [10,11,12,13].

However, accurately quantifying these cascade effects remains a significant challenge. Failure to account for them leads to substantial underestimation of mesozooplankton grazing rates [14,15], sometimes even yielding negative values when indirect effects outweigh direct grazing [16,17]. This gap distorts our understanding of planktonic energy transfer and responses to environmental forcing. Pioneering attempts to quantify these effects [14,15,18] have established the feasibility of measuring cascade strength, yet research remains limited, underscoring the need for a deeper investigation into the mechanisms and climate responsiveness of mesozooplankton-mediated trophic cascades (MMTC).

The urgency of this research is amplified by anthropogenic global change [11], which poses a globally pervasive threat to pelagic ecosystem stability and biogeochemical cycling by reshaping trophic interactions across oceanic realms from polar seas to tropical oligotrophic waters, and from coastal eutrophic systems to open ocean gyres. Ocean warming, acidification, and eutrophication are rapidly altering marine environments, and these stressors rarely act in isolation. Instead, they form a complex “cocktail” of interacting drivers that can fundamentally reorganize trophic interactions [19] and disrupt the MMTC on a global scale. For instance, ocean warming may increase metabolic demands and modify prey selection patterns, while acidification can impair sensory cues critical for feeding behavior [10]. Multi-stressor experiments reveal that combined effects often deviate from single-factor predictions, leading to outcomes such as the decoupling of microzooplankton grazing from phytoplankton growth in marine ecosystems worldwide [20]. Regional anthropogenic disturbances (e.g., riverine nutrient inputs, coastal hypoxia) in coastal marine systems worldwide including European coastal lagoons, the subtropical South Atlantic, and tropical estuarine ecosystems across the Pacific [21,22,23], further interact with global climate drivers [24,25], exacerbating cascade disruption and threatening local fisheries and carbon sequestration processes across temperate, tropical and polar regions, highlighting the need to link regional case studies to a global understanding of MMTC dynamics for cross-regional ecological prediction and management.

This review synthesizes current knowledge regarding mesozooplankton-driven trophic cascades. We first explore modern theoretical frameworks and mechanistic underpinnings of selective feeding. We then examine cascade dynamics across distinct oceanic realms and their ecosystem-level consequences. Finally, we assess how global change stressors modulate these interactions and propose future research directions to predict and manage marine ecosystem resilience under a changing climate.

## 2. Theoretical Foundations and Mechanistic Drivers

### 2.1. From Linear Cascades to Network-Based Interactions

The classic trophic cascade paradigm, largely derived from limnetic studies, posits an inverse relationship between biomass of alternating trophic levels (e.g., increased predator biomass → decreased herbivore biomass → increased producer biomass). While foundational, this model proves insufficient for marine systems characterized by high trophic connectivity, widespread omnivory, and prominent microbial loop dynamics [1,26]. Modern ecological theory embraces a network perspective wherein “interactive top-down and bottom-up control” acknowledges that cascade strength is simultaneously modulated by resource availability (bottom-up forces) and predation pressure (top-down forces). The ubiquity of omnivorous mesozooplankton creates reticulate food webs where energy flows through multiple pathways, stabilizing systems against perturbations while complicating cascade predictions. This complexity necessitates a paradigm shift from viewing cascades as simple population-level phenomena to conceptualizing them as emergent properties of trait-mediated interactions within food webs. Recent advances in trait-based ecology reveal that mesozooplankton functional trait syndromes (morphological, physiological, behavioral) rather than mere biomass determine cascade intensity, with traits such as body size, feeding appendage structure, and metabolic rate forming key regulatory axes [27]. Banse [28] emphasized that the pivotal role of mesozooplankton in regulating ocean production stems from their capacity to integrate top-down and bottom-up signals via trait plasticity—a core premise of modern cascade theory.

### 2.2. Selective Feeding: The Behavioral Engine of Cascades

Selective feeding constitutes the key behavioral mechanism that determines cascade initiation, strength, and direction (Table 1). Recent methodological advances, particularly DNA metabarcoding of gut contents, have unveiled unprecedented detail regarding mesozooplankton dietary composition, revealing a continuum from obligate selectivity to trophic plasticity [27,29]. Kleppel [30] noted that calanoid copepods, dominant mesozooplankton taxa in most marine ecosystems, exhibit context-dependent dietary shifts that blur the boundaries between herbivory and omnivory, underscoring the need to move beyond simplified feeding-guild classifications.

Size- and Motility-Based Selection: The physical constraints of feeding appendages establish a baseline size preference. However, prey selection is an active process; copepods such as *Temora longicornis* can exhibit consistent selection for specific prey taxa, such as *Synechococcales cyanobacteria*, across heterogeneous environments. Conversely, species including *Centropages hamatus* and *Acartia* spp. display dietary plasticity, adjusting their feeding preferences opportunistically in response to local prey availability. Motile prey, such as ciliates and dinoflagellates, often elicit stronger predatory responses than non-motile phytoplankton, attributable to hydrodynamic or chemical signaling cues [31]. Lombard et al. [32] demonstrated active prey rejection in appendicularians, a group of gelatinous mesozooplankton, highlighting that selective feeding is not restricted to copepods. Morphological traits play a pivotal role in this process—marine cladocerans (e.g., *Penilia avirostris*) exhibit a preference for picophytoplankton, while most copepods (e.g., *Acartia* and *Paracalanus*) efficiently graze on nanophytoplankton and microphytoplankton [33]. A study further illustrated that a shift from large to small mesozooplankton body size reduced phytoplankton grazing pressure by 40%, underscoring the intimate link between size structure and cascade dynamics [34]. Basedow et al. [34] applied the biovolume spectrum theories to mesozooplankton communities at the polar front, confirming that size-based trophic partitioning serves as a universal driver of cascade dynamics across latitudinal gradients.

Stoichiometric and Nutritional Quality Drivers: Beyond size, the elemental and biochemical composition of zooplankton is strongly influenced by their dietary intake (“you are what you eat”). To maintain optimal somatic growth and reproductive output, mesozooplankton selectively graze on prey to balance the acquisition of essential elements (e.g., nitrogen, phosphorus) for cellular stoichiometry and critical biochemicals (e.g., amino acids, polyunsaturated fatty acids [PUFAs]). For example, dinoflagellates and ciliates, which are typically rich in PUFAs and proteins, are often preferred over nutritionally inferior prey, even when the latter is more abundant. This “compensatory feeding” strategy under nutrient-imbalanced conditions represents a powerful driver of prey community structure [35]. Broglio et al. [36] found that the copepod *Acartia tonsa* achieves higher reproductive success when fed heterotrophic prey (ciliates) than when fed autotrophic prey, attributable to a superior fatty acid composition. Physiological traits such as metabolic rate and nutrient excretion ratio (N/P) further modulate this process—copepods can adjust their excretion N/P in response to food quality, buffering cascade fluctuations induced by nutrient imbalance [37]. Kattner et al. [38] provided a comprehensive overview of marine zooplankton lipids, emphasizing their role as key mediators of nutritional selectivity and cascade dynamics.

Toxicity Avoidance and Mixotrophy: The proliferation of harmful algal blooms (HABs) poses a significant challenge to trophic dynamics. Many mesozooplankton taxa actively avoid toxic phytoplankton species, a behavior that can disrupt the grazing link and facilitate bloom persistence [39]. Chen et al. [17] experimentally confirmed that mesozooplankton feeding selectivity can suppress non-toxic algae while enabling the proliferation of toxic strains, thereby altering the cascade’s direction. Furthermore, the increasing recognition of mixotrophy, wherein organisms integrate photosynthesis and phagotrophy, adds an additional layer of complexity to trophic interactions. Mixotrophic protists can function as both primary producers and consumers, blurring traditional trophic boundaries. Their feeding behavior, such as that observed in *Lepidodinium* sp., is highly sensitive to temperature and nutrient ratios, potentially exacerbating selective pressures under climate change [40]. Sommer [3] highlighted that mixotrophy modifies cascade pathways by creating “trophic shortcuts” that bypass conventional mesozooplankton-microzooplankton interactions.

Gelatinous Zooplankton-Specific Mechanisms: Gelatinous mesozooplankton (e.g., *Dolioletta gegenbauri*, doliolids) exhibit unique selective feeding strategies that contribute to trophic cascades via the “jelly pump”—a distinct vertical carbon export pathway. These taxa produce abundant mucous aggregates and fast-sinking fecal pellets, and recent studies have confirmed their role in channeling carbon from the microbial loop to deep-sea environments. Turner [41] emphasized that small gelatinous zooplankton and copepods are critical to the function of the pelagic food web due to their high abundance and their capacity to transfer energy from the microbial loop to higher trophic levels. This function amplifies the cascade effects on carbon cycling.

**Table 1:** Key mechanisms of mesozooplankton selective feeding and their cascade implications.

Selection Mechanism	Driver	Typical Prey Preference	Potential Cascade Effect
Size/Motility	Physical feeding structures, hydrodynamic detection	Larger, motile prey (ciliates, dinoflagellates) over small pico-phytoplankton	Suppression of microzooplankton, indirect enhancement of pico-phytoplankton biomass [31].
Stoichiometric (Elemental)	Homeostatic maintenance of cellular N/P ratios	Prey with N/P ratios approximating the consumer’s physiological requirement (often N-rich prey)	Alters nutrient recycling patterns, favoring phytoplankton with complementary stoichiometric profiles [37].
Nutritional (Biochemical)	Demand for essential fatty acids (e.g., DHA, EPA) and amino acids	Prey rich in high-quality lipids and proteins (e.g., certain dinoflagellate taxa)	Shifts prey community toward high-nutritional-quality taxa, potentially enhancing mesozooplankton fitness and secondary production [35].
Toxin Avoidance	Detoxification costs, behavioral rejection of toxic taxa	Non-toxic over toxic phytoplankton (e.g., non-PSA-producing over PSA-producing dinoflagellates)	Decouples grazing pressure from phytoplankton biomass, potentially facilitating the proliferation of toxic HAB species [39].

### 2.3. Fundamental Concepts and Pathways

**Figure 1:**
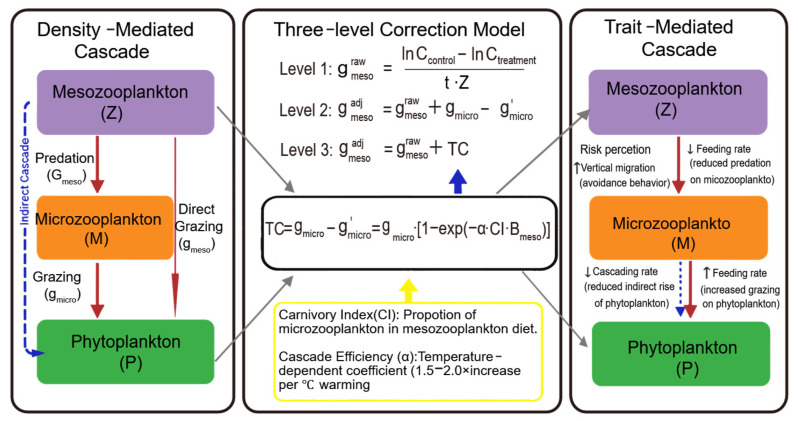
Dual–pathway regulatory framework of mesozooplankton-mediated trophic cascades.

Trophic cascades refer to ecological processes wherein predators indirectly promote primary producer biomass by suppressing herbivore populations [1,26,42]. Paine [42] laid the theoretical groundwork for understanding food web connectivity and interaction strength, which are essential for quantifying cascade effects. In marine pelagic ecosystems, mesozooplankton-mediated trophic cascades (MMTC) operate through two primary pathways (Figure 1):

Pathway 1: Density-Mediated Cascade (DMC): Mesozooplankton reduce microzooplankton population density via direct predation, thereby decreasing microzooplankton grazing pressure on phytoplankton (g) and increasing phytoplankton biomass. The strength of this pathway is quantified as follows:

$$DMC=\frac{\Delta Phytoplankton\;Biomass}{\Delta Mesozooplankton\;Predation\;Pressure}$$

Pathway 2: Trait-Mediated Cascade (TMC): Mesoooplankton modify their feeding behavior in response to prey availability and predator risk, which has implications for both predator and prey populations [43]. Predation risk induces behavioral modifications in mesozooplankton (e.g., copepod diel vertical migration, altered feeding periodicity), reducing their effective grazing rate even in the absence of changes in population density [35,44]. This pathway is particularly prominent in turbulent mixed layers, where microhabitat heterogeneity further modulates behavioral responses [44].

### 2.4. Three-Level Grazing Rate Correction Model

Traditional grazing rate estimation methods are plagued by three systematic biases. We propose a progressive correction framework to address the long-standing underestimation of mesozooplankton grazing pressure:

Level 1—Basic Frost’s Clearance Rate Method [45]: The initial calculation of mesozooplankton grazing rate without accounting for microzooplankton interference or cascade effects is as follows:

$$g_{meso}^{raw}=\frac{\ln\left(\frac{C_{control}}{C_{treatment}}\right)}{t\;\cdot \;Z}$$

where $C_{\mathrm{control}}$ and $C_{\mathrm{treatment}}$ represent phytoplankton concentrations in the control and mesozooplankton-containing groups, respectively. t denotes incubation time, and Z is mesozooplankton abundance or biomass [14,15,45]. Nejstgaard et al. [15] noted that this method inherently underestimates microzooplankton grazing interference, highlighting the necessity of subsequent corrections. 

Level 2—Microzooplankton Background Grazing Subtraction [27]: Adjustment for inherent grazing pressure exerted by microzooplankton in the study system is as follows:

$$g_{meso}^{adj}=g_{meso}^{raw}+g_{micro}-{g^{\prime }}_{micro}$$

where $g_{micro}$ and ${g^{\prime }}_{micro}$ are the measured microzooplankton grazing rate in the absence and presence of mesozooplankton, respectively [7,46]. Calbet [46] conducted a global comparative analysis, confirming that microzooplankton background grazing accounts for 30–70% of phytoplankton mortality in most marine ecosystems, rendering this correction indispensable.

Level 3—Trophic Cascade Dynamic Compensation (Core Innovation): Incorporation of indirect cascade effects on microzooplankton grazing rates, the rate of adjusted mesozooplankton grazing rate on phytoplankton will be as follows:

$$g_{meso}^{adj}=g_{meso}^{raw}+TC$$

and the rate of trophic cascades will be as follows:

$$TC=g_{micro}-g_{micro}^{\prime }=g_{micro}\times \left[1-exp\left(-\alpha \cdot CI\cdot B_{meso}\right)\right]$$

where α represents the cascade efficiency coefficient (increasing 1.5–2.0 fold with rising temperature), CI denotes the mesozooplankton carnivory index (proportion of microzooplankton in the diet), and $B_{meso}$ is mesozooplankton biomass. Gifford et al. [47] validated the relationship between the carnivory index and cascade strength in estuarine systems, supporting the inclusion of CI as a key parameter.

### 2.5. Quantification of Cascade Strength (CS)

**Figure 2:**
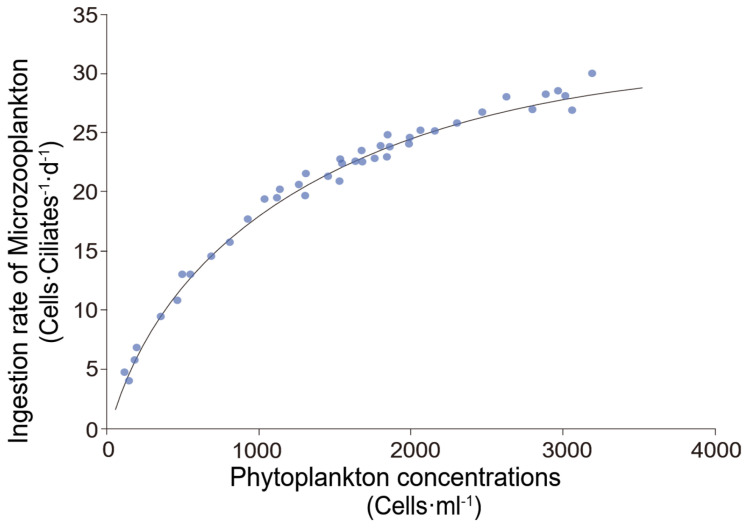
Functional response curve of feeding rate of microzooplankton to phytoplankton concentrations (Modified from Chen et al., [17]). The points are fitted by the Michaelis–Menten equation by $I=I_{max}\times x/(k_{d}+x)$. For example, $I_{\max}$ and $k_{d}$ are calculated as 38 cells ciliates^−1^ d^−1^ and 1093 cells mL^−1^, respectively, in Chen et al. [17].

Chen et al. [14,17] provided another practical way to estimate $g_{micro}$, as the feeding rate of microzooplankton exhibits a functional response to food concentration. When the food concentration reaches a certain threshold, the feeding rate of microzooplankton becomes saturated. The regression relationship between them can be fitted using the Michaelis–Menten equation. Taking the model of ciliate ingestion rate vs. green algae concentration fitted by Chen et al. [17] as an example (Figure 2), the functional relationship between ciliate feeding rate and food concentration can be expressed as follows:

$$I\left(x\right)=I_{max}\times x/(k_{d}+x)$$

where I(x) is the feeding rate of ciliates at food concentration x (a function of x), $I_{max}$ is the maximum individual feeding rate achievable by ciliates, and $k_{d}$ is the food concentration at which half of $I_{max}$ is attained. Meanwhile, since the feeding rate of microzooplankton is derived from the clearance rate ($F_{micro}$) and ingestion rate ($I_{micro}$), then the rate can be estimated by the following:

$$I_{micro}=F_{micro}\times P_{mean}=g_{micro}/M_{mean}\times P_{mean}$$

$$g_{micro}=M_{mean}\times I_{micro}/P_{mean}$$

where $M_{mean}$ and $P_{mean}$ are the average concentrations of microzooplankton and phytoplankton during the culture period, respectively. Substituting the formula of I(x) into the equation of $g_{micro}$ yields the two-factor regression relationship between the microzooplankton ingestion rate g(x,y) and the average concentrations of food (x) and grazers (y):

$$g\left(x,y\right)=\frac{y}{x}\times I_{max}\times \frac{x}{k_{d}+x}=y\times I_{max}/(k_{d}+x)$$

In summary, the basic workflow for measuring and calculating trophic cascade effects is as follows: (1) Establishing the regression relationship between microzooplankton ingestion rate ($I_{micro}$, obtained from the dilution method) and microzooplankton biomass or abundance ; (2) Estimating the microzooplankton grazing rate g(x,y) in both the mesozooplankton grazing experiment treatment group and the control group based on the regression relationship, respectively; (3) Estimating the strength of the trophic cascade effect (TC) by the difference in microzooplankton grazing rate ($g_{micro}-{g^{\prime }}_{micro}$) between the treatment group and the control group. 

In this review, we propose quantifying cascade effects using the Cascade Strength index (CS), which comprehensively integrates absolute cascade-induced changes and relative biomass structure:

$$CS=\frac{g_{micro}\;-g_{micro}^{\prime }}{g_{micro}}\times \ln\left(\frac{B_{meso}}{B_{micro}}\right)$$

where $g_{micro}$ represents the microzooplankton grazing rate in the control group (without mesozooplankton), ${g^{\prime }}_{micro}$ denotes the grazing rate in the presence of mesozooplankton, and B signifies the biomass of the respective trophic groups. This formula effectively reflects the indirect promotional effect of mesozooplankton on phytoplankton via microzooplankton suppression, with CS > 0 indicating a positive cascade effect (mesozooplankton enhances phytoplankton biomass), CS = 0 indicating the absence of a cascade effect, and CS < 0 indicating an inverse cascade effect.

### 2.6. NPMZ Model: Integrating Mesozooplankton-Microzooplankton-Phytoplankton Interactions

To quantitatively predict mesozooplankton-mediated trophic cascade (MMTC) dynamics and to validate the three-level grazing rate correction model, the traditional NPZ (Nutrient–Phytoplankton–Zooplankton) model requires extension through the incorporation of microzooplankton (M) as a key intermediate trophic level, forming the NPMZ model. This model explicitly simulates the dual role of mesozooplankton (Z) as grazers of both phytoplankton (P) and microzooplankton, as well as the reciprocal trophic interactions between microzooplankton and phytoplankton, thereby addressing the limitation of traditional models that overlook microbial loop processes [48,49,50]. Buitenhuis et al. [48] emphasized that biogeochemical fluxes mediated by mesozooplankton cannot be accurately modeled without accounting for microzooplankton interactions, underscoring the necessity of the NPMZ framework. The model structure is calibrated using global field observation data and laboratory experimental results, enabling its application across diverse marine environments from coastal waters to the open ocean.

The core differential equations of the NPMZ model are presented below, describing the temporal dynamics of nutrient (*N*), phytoplankton (*P*), microzooplankton (*M*), and mesozooplankton (*Z*) biomass (units: mg C·L^−1^·d^−1^), with strict adherence to mass conservation principles:

 dNdt=−rmaxNPkN+N+lPNP+lMNM+lZNZ+γMgMM+γZ(gZ+GZ)Z

dPdt=rmaxNPkN+N−lPNP−gMM−gZZ

dMdt=(1−γM)gMM−lMNM−GZZ

  dZdt=(1−γZ)(gZ+GZ)Z−lZNZ

dNdt+dPdt+dMdt+dZdt=0

where $r_{\max}$ denotes the maximum phytoplankton growth rate (following Michaelis-Menten kinetics); $k_{N}$ is the half-saturation constant for phytoplankton nutrient uptake; $l_{\mathrm{PN}}$, $l_{\mathrm{MN}}$, $l_{\mathrm{ZN}}$ represent the mortality/mineralization rates of phytoplankton, microzooplankton, and mesozooplankton, respectively; $\gamma_{M}$,$\gamma_{Z}$ are the non-assimilated fractions of prey by microzooplankton and mesozooplankton (ranging from 0.2 to 0.4, dimensionless); $g_{M}$ is the microzooplankton grazing rate on phytoplankton; $g_{Z}$ is the mesozooplankton grazing rate on phytoplankton; and $G_{Z}$ is the mesozooplankton grazing rate on microzooplankton. Chen et al. [51] experimentally validated the interaction terms between omnivorous copepods, heterotrophic dinoflagellates, and diatoms, providing empirical support for the model’s trophic linkages. The mass conservation equation ensures that the total material within the system remains balanced, consistent with the fundamental biogeochemical principles.

Model parameterization is based on a synthesis of global studies, with parameter ranges and corresponding references presented in Table 2. Sensitivity analysis indicates that $G_{Z}$ (mesozooplankton grazing rate on microzooplankton) and $\gamma_{Z}$ (mesozooplankton assimilation efficiency) are the most influential parameters regulating cascade strength. This aligns with the core logic of the three-level grazing rate correction model: mesozooplankton predation on microzooplankton drives the indirect cascade [26,51]. Chen et al. [18] demonstrated that dietary essential fatty acids influence copepod reproduction rates, which in turn modulate $\gamma_{Ζ}$ and cascade dynamics.

The NPMZ model provides a quantitative framework for exploring MMTC mechanisms. For example, by simulating the response of $G_{Z}$ to temperature changes, the model can predict how warming alters cascade strength [53]. For instance, mechanistic ecological models incorporating temperature-dependent physiological rates predict that an increase in sea surface temperature (SST) of 2 °C leads to a $G_{Z}$ increase of 0.15–0.2 d^−1^, resulting in a 15–20% enhancement in cascade intensity due to temperature-driven shifts in omnivorous copepod diet preferences (increase carnivory at elevated temperatures) [54]. This prediction aligns with field observations in certain regions, suggesting that warming generally strengthens top-down control in marine ecosystems [55,56]. Future model improvements should focus on incorporating trait-based parameters (e.g., mesozooplankton body size, feeding appendage structure) and multi-stressor interactions, further enhancing its capacity to predict MMTC dynamics under global change.

## 3. Dynamics and Manifestations of Trophic Cascades

### 3.1. Physical Forcing Mechanisms and Biotic Feedback Loops

Abiotic oceanographic processes modulate MMTC dynamics by altering mesozooplankton distribution, prey availability, and trophic interactions [25]. Mixed layer depth (MLD) is a key regulator—shallow MLD enhances prey encounter rates, strengthening cascade intensity by 15–25% compared to deep mixed layers, where mesozooplankton and prey are vertically dispersed [25]. In addition, vertical mixing can enhance nutrient supply and disrupt grazer-phytoplankton synchrony, thereby affecting trophic interactions [57]. Seasonal deepening of the MLD reduces mesozooplankton predation on microzooplankton, weakening cascade effects and shifting phytoplankton community structure toward smaller taxa [58]. 

Ocean currents and eddies further modify spatiotemporal patterns: anticyclonic eddies concentrate nutrients and prey, fostering higher mesozooplankton biomass and *CS* values [59], while cyclonic eddies disperse communities, dampening cascade strength [60,61]. For example, Gangrade and Mangolte [62] reported that the transport of plankton by dynamic mesoscale currents shapes the structure of plankton communities in the California Current System (CCS). Anticyclonic eddies concentrate nutrients and prey, increasing mesozooplankton biomass and cascade strength (*CS*) values, while cyclonic eddies disperse communities and dampen cascades [60,61].

Upwelling events in coastal systems deliver nutrient-rich deep water, stimulating phytoplankton blooms [42] and altering mesozooplankton diet preferences by switching from microzooplankton to phytoplankton grazing [63,64]. This diet switching, from a microzooplankton-dominated diet to a more direct phytoplankton-dominated diet, is a critical adaptation to capitalize on the increased primary productivity. When mesozooplankton directly graze phytoplankton due to upwelling—induced blooms, their reliance on microzooplankton as prey diminishes. This reduction in predation pressure on microzooplankton can allow microzooplankton populations to increase or maintain their biomass, even as phytoplankton are heavily grazed by mesozooplankton. Consequently, the top-down control exerted by mesozooplankton on microzooplankton is dampened, thereby weakening the indirect cascading effects on phytoplankton [65]. Studies have shown that feeding behavior can be context-dependent [66]. For example, Wan et al. [23] provided mechanistic evidence by analyzing gut fluorescence, fatty acid and elemental compositions, and stable isotope ratios of mesozooplankton in the Humboldt Current Upwelling System, confirming rapid diet reconfiguration within days of upwelling onset. García-Seoane et al. [64] also observed a shift in mesozooplankton diet from microzooplankton to phytoplankton in the coastal system off NW Iberian Peninsula, showing a dampened MMTC.

Biotic Feedback Loops regulate MMTC stability, creating reciprocal interactions between trophic levels. Positive feedback loops can amplify cascade effects: for example, enhanced phytoplankton biomass from weak microzooplankton grazing provides more food for mesozooplankton, which in turn increases their predation on microzooplankton, further strengthening the cascade [14,67]. Empirical evidence from the Pearl River Estuary confirms that mesozooplankton exert strong size- and taxon-selective grazing on microzooplankton, thereby indirectly releasing phytoplankton from grazing pressure and amplifying bloom formation [14]. Such self-reinforcing dynamics align with theoretical predictions of “trophic amplification,” wherein perturbations at higher trophic levels propagate disproportionately through food webs—a phenomenon observed globally under climate change scenarios, where marine biomass declines accelerate with trophic level [68]. In contrast, negative feedback loops buffer against extreme fluctuations: if mesozooplankton predation reduces microzooplankton biomass too severely, mesozooplankton switch to phytoplankton grazing, particularly larger diatoms and colonial forms, thereby dampening the indirect phytoplankton enhancement that defines the classic cascade [33]. This behavioral plasticity has been empirically documented in the Amundsen Sea polynya, where copepod ingestion rates on phytoplankton increased significantly during late summer when microzooplankton prey densities declined [67]. Stoichiometric feedbacks also play a role—nutrient excretion by mesozooplankton (adjusted for prey quality) modulates phytoplankton growth [20,52]. Critically, this process creates a feedback loop: phytoplankton community shifts induced by excretion alter the stoichiometric quality of the food available to microzooplankton, which, in turn, modulates their growth, grazing efficiency, and susceptibility to mesozooplankton predation [20,59]. The integration of these three feedback modalities explains why mesozooplankton trophic cascades are neither universally strong nor uniformly predictable. Field studies reveal substantial spatial and temporal heterogeneity in cascade strength—ranging from strong top–down control in oligotrophic Gulf of Mexico bluefin tuna spawning habitats to weak or negligible effects in highly eutrophic estuaries like Xiangshan Bay, where bottom-up forcing dominates [69,70].

**Figure 3:**
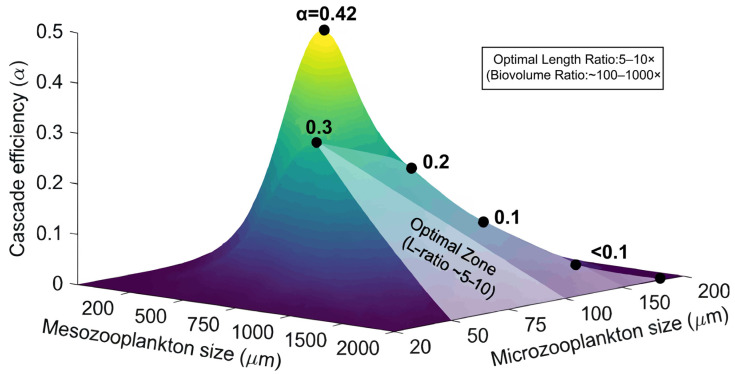
Schematic diagram illustrating the effects of predator–prey size matching on trophic cascade efficiency. The 3D surface illustrates the relationship between mesozooplankton (200–2000 μm) and microzooplankton (20–200 μm) size fractions and the resulting cascade efficiency coefficient (α). A pronounced peak in efficiency (α ≈ 0.42) occurs when the predator is approximately 10 times larger than its prey (e.g., 1000 μm vs. 100 μm). Secondly, lower peaks align with size ratios between 5:1 and 10:1, forming an optimal efficiency ridge along the diagonal. Marked efficiency declines outside this ratio highlight that size mismatch can reduce cascade strength by >50%. This pattern underscores the importance of optimal body-size scaling (length ratio ~5–10; biovolume ratio ~100–1000) in mediating strong top-down control in planktonic systems.

This size relationship critically determines the intensity of predator-prey interactions and, thus the size matching between mesozooplankton predators and their microzooplankton prey significantly influence the strength of trophic cascades [14]. The effect of size matching between mesozooplankton and microzooplankton on cascade efficiency in illustrated in Figure 3. Optimal size matching occurs when mesozooplankton are approximately 5 to 10 times larger in body length than their microzooplankton prey, which translates to a biovolume ratio of about 100 to 1000 times [71].Within this optimal range, mesozooplankton exhibit maximal ingestion rates and high digestion efficiency, leading to a more pronounced population-level impact on microzooplankton and a stronger trophic cascade [14]. Deviations from this optimal size ratio can reduce cascade strength. If mesozooplankton are undersized, meaning their body length is less than approximately five times that of their microzooplankton prey, they may struggle with capture success and handling efficiency, particularly when preying on larger microzooplankton species such as tintinnids or ciliates that are greater than 80 µm [71]. Conversely, if predators are oversized, exceeding a 15-fold length ratio, their functional responses might be reduced due to energetic inefficiencies. In such cases, these larger mesozooplankton may shift their selectivity towards smaller, more abundant microzooplankton, such as heterotrophic nanoflagellates, potentially altering the cascade’s overall effectiveness [14,16]. Changes in phytoplankton size-structure also impact, with smaller phytoplankton dominating in summer and larger cells in winter, affecting grazing dynamics [58].

### 3.2. Spatiotemporal Heterogeneity

Trophic cascades exhibit significant spatiotemporal heterogeneity in marine systems, largely influenced by physical oceanographic processes [6,57,72]. This variability is shaped by a suite of factors, including nutrient supply, light availability, plankton community size structure, predator-prey size matching, stoichiometric quality, temperature, and anthropogenic disturbance intensity [14,71,73,74]. Regionally, contrasting patterns are observed across marine ecosystems, with each realm characterized by unique heterogenic traits linked to environmental and biological filters:

Eutrophic Coastal/Estuarine Systems: Eutrophic coastal and estuarine systems are defined by high nutrient loading, strong physical mixing, and frequent anthropogenic disturbance (eutrophication, hypoxia), leading to high temporal and small-scale spatial heterogeneity in MMTC [21,23]. High nutrient loads support large phytoplankton biomass and diverse plankton communities, where mesozooplankton exert both direct top-down control on phytoplankton and indirect control via predation on microzooplankton, creating context-dependent directionality [16,74,75]. A key heterogenic characteristic of these systems is the shift between bottom-up and top-down forcing driven by anthropogenic stressors: overfishing-induced mesopredator release weakens top-down control on mesozooplankton [23,25,52], while hypoxia, on the other hand, reduces mesozooplankton predation efficiency on microzooplankton, dampening the indirect cascade effects in oxygen-poor hotspots [75,76]. For example, in temperate estuaries such as Chesapeake Bay and the northern Gulf of Mexico, seasonal hypoxia and freshwater inflow modulate mesozooplankton community composition and grazing pressure, leading to pronounced interannual variability in cascade intensity [66,77]. In Chinese coastal systems, comparative studies reveal contrasting patterns: while Xiangshan Bay exhibits weak trophic cascades due to dominance of bottom-up forcing [69], Daya Bay shows moderate cascade effects modulated by phytoplankton size structure and temperature [74,78]. European estuaries such as the Scheldt estuary and Neva estuary further demonstrate that anthropogenic nutrient loading interacts with tidal dynamics to alter zooplankton functional diversity and trophic coupling [79,80,81]. These examples underscore that eutrophic systems are not uniform; rather, cascade strength reflects the balance between nutrient loading, grazing pressure, and fisheries impacts. This highlights how the interplay between biotic interactions and abiotic factors determines the strength of cascades even in productive coastal systems.

Oligotrophic Open Oceans: These regions (e.g., subtropical North Pacific) are characterized by low nutrient availability, stable physical conditions, and dominance of picophytoplankton (<2 µm), leading to low temporal variability but strong vertical spatial heterogeneity in MMTC [7,70,82]. Unlike coastal systems, MMTC in open oceans is rarely disrupted by anthropogenic stressors, leading to long-term stability in cascade direction [12]. A core heterogenic trait of these systems is the reliance of mesozooplankton on microzooplankton as a primary food source (contributing > 60% to dietary intake), amplifying indirect cascade effects that promote picophytoplankton dominance by relieving microzooplankton grazing pressure [7,46]. The vertical heterogeneity of plankton structure in open oceans is primarily driven by mixed layer depth (MLD) and mesoscale eddies, as mentioned above. For example, in the North Pacific Subtropical Gyre, seasonal variability in mixed layer depth modulates prey availability and cascade strength [83,84]. In contrast, the Mediterranean Sea exhibits stronger cascades in its western basin than in its eastern basin, attributed to differences in nutrient availability and zooplankton community structure [85,86]. Eddy-induced nutrient pulses in oligotrophic waters can temporarily enhance primary production and alter trophic pathways, as demonstrated in many areas, such as the western equatorial Pacific Ocean [60], the Oligotrophic North Atlantic [87], the South China Sea [88], etc. 

Polar Marine Ecosystems: These regions are particularly sensitive to climate change, exhibiting distinct patterns of trophic cascades [89]. Warming temperatures, freshening seawater, and disruptions to sea ice formation significantly impact the distribution, abundance, and structure of zooplankton assemblages, which are crucial for trophodynamics [90]. Seasonal sea ice loss can alter light and nutrient phenology, compressing phytoplankton blooms and shifting zooplankton phenology, potentially decoupling predator-prey synchrony [91]. It is hypothesized that cascades may strengthen under ice retreat if apex predator recovery suppresses krill or forage fish consumers, though evidence remains highly ecosystem-specific and context-dependent [91]. Generally, Polar regions (>60° N/S) exhibit high trophic cascades values, primarily attributable to high microzooplankton biomass and grazing pressure, coupled with the dominance of large-bodied mesozooplankton taxa (e.g., *Calanus propinquus*) with high carnivory indices, triggering strong indirect enhancement of phytoplankton biomass adapted to polar light and nutrient regimes [65,92]. Yet, pronounced heterogeneity arises from sea ice dynamics, light regimes, and regional oceanography. In the Arctic, cascade strength varies between the Barents Sea, where Atlantic water inflow sustains high zooplankton production [93], and the Chukchi Sea and Canada Basin, where sea ice retreat timing controls phenological match-mismatch dynamics [94,95]. In the Antarctic, contrasting patterns emerge between the Weddell and Ross Seas, driven by differences in iron limitation and krill grazing pressure [96,97]. Climate-induced sea ice loss is reshaping these systems unevenly: while some regions experience strengthened cascades due to prolonged open-water seasons, others face trophic decoupling as zooplankton phenology diverges from phytoplankton blooms [91,92]. This regional variability underscores the need for continued monitoring across polar habitats.

**Figure 4:**
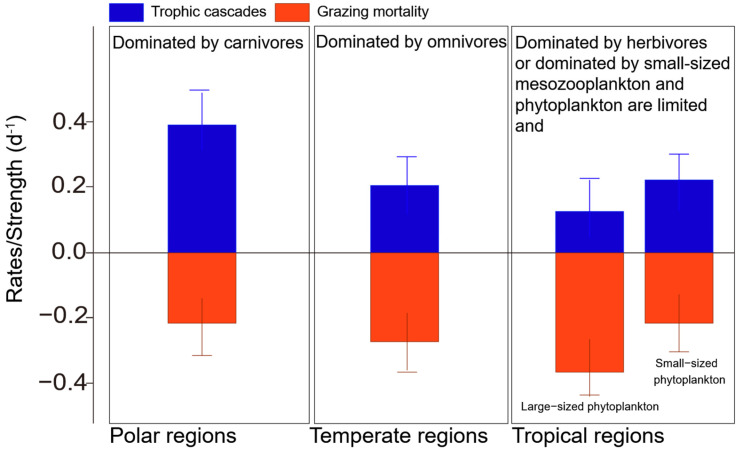
Effect of different mesozooplankton communities on phytoplankton, expressed as the rate of algal growth or the strength of cascades (reprocessed and extrapolated from Chen et al., 2013 [17]). Grazing mortality (red bars) caused the decrease in algal growth rate, while cascading effects (trophic cascades) induced by feeding on intermediate grazers (blue bars) caused the increase in algae. The data presented are not actual measurements from polar, temperate, and tropical regions, but were derived by extrapolating these relationships to polar/temperate/tropical marine systems based on the typical mesozooplankton community traits and plankton trophic structure characteristics of each zone (Polar: Basedow et al., [34]; Rakowski et al. [65]; Temperate: Chen et al. [74]; Cabrerizo et al. [73]; Tropical: Calbet & Landry [7]; Landry et al. [82]).

Latitudinal and Cross-System Synthesis: The existence and consistency of latitudinal gradients in MMTC strength represent a key global heterogenic feature, with patterns modulated by latitudinal changes in mesozooplankton body size, plankton community structure, and environmental conditions [73,98,99]. Generally, cascade strength typically peaks in polar systems, where large-bodied copepods exert strong top-down control, as mentioned above. Temperate regions (30–60° N/S) generally display intermediate CS values, driven by seasonal shifts in mesozooplankton functional traits and prey availability [73]. Studies in the East China Sea showed that a higher proportion of large phytoplankton, higher stoichiometric quality of phytoplankton, and higher temperature could mitigate the intensity of copepod–induced trophic cascades [74]. Tropical regions (<30° N/S) exhibit the lowest CS values, constrained by nutrient limitation, small-bodied mesozooplankton dominance, and high picophytoplankton abundance [7,82]. The comparative patterns of mesozooplankton community-driven cascade strength across polar, temperate, and tropical zones, and their differential effects on phytoplankton growth rates, are further visualized in Figure 4. Latitudinal gradient heterogeneity is further modified by anthropogenic stressors: fishing pressure and coastal eutrophication reduce the strength of cascades in temperate and tropical coastal systems, blurring the natural latitudinal pattern [6,99].

Collectively, spatiotemporal heterogeneity in MMTC is a multifaceted feature shaped by the interactions among physical oceanographic processes, biotic community traits, and anthropogenic disturbances, with each marine ecosystem realm exhibiting unique heterogeneity. Eutrophic coastal/estuarine systems are defined by high temporal and small-scale spatial heterogeneity driven by anthropogenic stressors and physical mixing; oligotrophic open oceans display low temporal variability but strong vertical spatial heterogeneity linked to MLD and mesoscale eddies; polar ecosystems exhibit extreme seasonal heterogeneity coupled to sea ice dynamics; and latitudinal gradients reflect systematic changes in cascade strength driven by mesozooplankton body size and plankton community structure.

### 3.3. Temporal Dynamics of Trophic Cascades

MMTC exhibits complex temporal dynamics, with short-term (diel and weekly) and long-term (seasonal and interannual) fluctuations modulated by biotic feedback loops. Diel Vertical Migration (DVM) of mesozooplankton and microzooplankton creates diel variability in cascade strength [2]. During daylight hours, mesozooplankton migrate to deeper, darker waters to avoid visual predators, reducing predation pressure on surface-dwelling microzooplankton and weakening cascade effects [100]. At night, mesozooplankton return to the euphotic zone, intensifying predation on microzooplankton and strengthening cascades—this diel shift can alter CS values by 10–15% [101]. In turbulent mixed layers, DVM is suppressed, leading to consistent daytime predation and stronger, more stable cascades [79].

Seasonal Succession drives predictable shifts in MMTC intensity, tightly coupled to phytoplankton bloom dynamics and mesozooplankton life cycles [102]. In temperate systems, spring diatom blooms provide abundant food for mesozooplankton, triggering reproductive peaks and increased predation on microzooplankton—cascade strength peaks during bloom decline, when mesozooplankton biomass is high, and microzooplankton are abundant [103]. Summer stratification reduces nutrient availability, shifting phytoplankton dominance toward smaller taxa and altering mesozooplankton diet preferences, thereby reducing cascade intensity [103,104]. Winter mixing resets the system, with low mesozooplankton biomass minimizing predation pressure on microzooplankton and weakening cascades [105]. The seasonal variability of mesozooplankton feeding rates in subtropical coastal and estuarine waters also reflects these changes in phytoplankton assemblages [14,16].

### 3.4. Ecosystem-Level Consequences of MMTC

Mesozooplankton-mediated trophic cascades exert profound impacts on pelagic ecosystem structure and function, with implications for biogeochemical cycling, secondary production, and fisheries sustainability. These cascades are complex interactions where changes at one trophic level propagate through the food web, often affecting non-adjacent levels.

Plankton Community Size Structure is a primary target of MMTC—selective mesozooplankton predation on microzooplankton indirectly promotes the dominance of smaller phytoplankton (picophytoplankton, nanophytoplankton) in oligotrophic systems, while in polar and temperate systems, it favors larger taxa (diatoms) by relieving microzooplankton grazing pressure [106,107]. In oligotrophic systems, this selective predation can indirectly lead to a dominance of smaller phytoplankton such as picophytoplankton and nanophytoplankton. Conversely, in polar and temperate systems, MMTC can favor larger phytoplankton taxa, such as diatoms, by alleviating microzooplankton grazing pressure. This size–structured response modulates the efficiency of the microbial loop [108,109,110]. When small phytoplankton dominate, carbon flow is enhanced through the microzooplankton–mesozooplankton pathway. In contrast, the dominance of larger phytoplankton promotes direct mesozooplankton grazing and faster sinking, altering vertical carbon export.

Biological Carbon Pump (BCP) Efficiency is tightly linked to MMTC dynamics [22,111]. In polar regions, strong cascades promote diatom blooms—diatoms produce large, fast-sinking aggregates and fecal pellets, enhancing vertical carbon flux by 40–60% compared to non-bloom periods [112,113]. Conversely, in tropical oligotrophic systems, weak cascades and picophytoplankton dominance reduce sinking flux, with most carbon recycled in the euphotic zone. Gelatinous mesozooplankton further modify BCP efficiency via the “jelly pump”: their mucous aggregates and fecal pellets sink rapidly, channeling carbon from the microbial loop to deep-sea environments [114]. For example, doliolid blooms, triggered by cascade effects in the northern South China Sea, have been observed to enhance vertical carbon flux by 28%, underscoring the important, often underappreciated, role of gelatinous taxa in cascade-mediated carbon sequestration [115].

Secondary Production and Fisheries Support are indirectly regulated by MMTC [97,116]. By altering the composition and nutritional quality of the phytoplankton community, these cascades directly influence the fitness and biomass of mesozooplankton, which are vital prey for commercially important fish species such as herring and sardines [117]. In temperate coastal systems, robust cascades can increase the availability of high-quality phytoplankton rich in polyunsaturated fatty acids (PUFAs), boosting mesozooplankton secondary production by 25–35% and supporting higher trophic levels. This highlights how the bottom-up effects of phytoplankton quality can propagate through the food web, impacting fish populations [117]. Conversely, cascade disruption by anthropogenic stressors (e.g., hypoxia, eutrophication) can reduce mesozooplankton quality and abundance [21,118], leading to trophic mismatches and declines in fish stocks. For example, in the East China Sea estuary, hypoxia-induced weakening of MMTC reduced mesozooplankton PUFA content, contributing to reduced growth rates in juvenile herring [119,120].

## 4. Impacts of Global Change on Mesozooplankton-Mediated Trophic Cascades

Global change significantly impacts Mesozooplankton-mediated Trophic Cascades (MMTC), which are crucial for energy transfer within pelagic food webs [121]. The cumulative effects of multiple anthropogenic stressors, such as acidification, eutrophication, and hypoxia, can profoundly alter these cascades, often with non-additive and complex consequences for marine ecosystems.

### 4.1. Ocean Warming

Ocean warming is a primary driver of MMTC disruption, altering mesozooplankton physiology, behavior, and trophic interactions [10,13]. Elevated SST increases mesozooplankton metabolic rates, raising their energy requirements and modifying prey selection patterns. Many taxa shift toward higher-quality prey (e.g., lipid-rich microzooplankton) to meet increased energy demands, strengthening cascade intensity in the short term [20]. However, the efficiency of energy transfer across trophic levels can decline with warming, as respiratory costs often increase more rapidly than energy intake, leading to a reduction in the Gross Growth Efficiency (GGE) of consumers [121]. However, long-term warming can reduce mesozooplankton biomass by increasing mortality and reducing reproductive success, weakening cascade effects despite enhanced per capita predation rates [122,123]. Furthermore, ocean warming contributes to increased warme and less dense surface waters form a barrier that suppresses vertical mixing and limits the resupply of essential nutrients from deeper waters to the euphotic zone.

**Figure 5:**
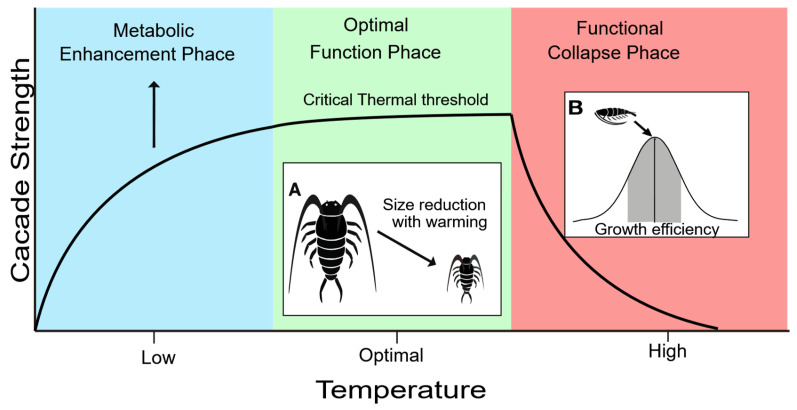
Conceptual model of the three-phase thermal regulation on mesozooplankton-mediated trophic cascade strength. This schematic illustrates how temperature nonlinearly modulates cascade strength (black curve). It identifies three distinct phases: (Low) a Metabolic Enhancement Phase at lower temperatures, typically occurs within a temperature range of approximately 15–20 °C, where cascade strength increases with warming; (Optimal) an Optimal Function Phase within a thermal window, observed roughly between 20 and 24 °C, where strength stabilizes at its maximum; and (High) a Functional Collapse Phase above a critical threshold (>24 °C), where strength declines precipitously. An inset highlights the concomitant decline in mesozooplankton body size with warming, a key trait-mediated mechanism. The model emphasizes that optimal cascade effects occur within a narrow thermal range, beyond which systemic efficiency collapses.

The impact of warming is not spatially uniform. For example, it exhibits pronounced latitudinal and regional variability. In tropical systems (e.g., the South China Sea), where ambient temperatures already approach or exceed thermal optima for many copepods, warming of just 1–2 °C can push communities into the “functional collapse phase” (Figure 5), leading to sharp declines in body size and feeding efficiency, and consequently, a rapid weakening of cascade strength [74]. In contrast, in subpolar regions (e.g., the Barents Sea), moderate warming can extend the productive growing season and enhance the dominance of large-bodied copepods like *Calanus finmarchicus*, potentially strengthening top-down control [104]

Furthermore, warming interacts with seasonal cycles. Warming alters the timing of mesozooplankton reproductive cycles and phytoplankton blooms, creating trophic mismatches. In temperate systems, warming can advance the spring phytoplankton bloom but delay zooplankton peak abundance, creating a temporal mismatch [103] that mesozooplankton reproductive peaks no longer coincide with microzooplankton abundance, predation pressure is reduced, and cascade strength declines [124,125]. Such mismatches can occur when different trophic levels respond to temperature cues at varying rates, leading to uncoupled life-history events [72].

### 4.2. Ocean Acidification

Ocean acidification (OA), driven by increased atmospheric CO_2_ absorption, impairs mesozooplankton sensory and behavioral traits, disrupting predator-prey interactions and MMTC dynamics [10]. OA reduces copepods’ ability to detect chemical cues from microzooplankton and toxic phytoplankton, altering selective feeding behavior. Copepods exhibit reduced avoidance of toxic taxa and decreased preference for high-quality microzooplankton, thereby weakening the directionality of the cascade [10]. OA also impacts mesozooplankton physiology by reducing growth rates and assimilation efficiency. Lower pH conditions can disrupt vital cellular processes, including protein synthesis and lipid metabolism, decreasing the ability of mesozooplankton to convert prey into biomass. This reduces mesozooplankton secondary production, even if prey availability remains unchanged, leading to weaker predation pressure on microzooplankton and diminished cascade effects. Notably, the effects of OA are not uniform and are notably context-dependent, often exacerbated by interactions with other environmental stressors such as ocean warming [10,13].

The spatiotemporal heterogeneity of OA effects is particularly evident when comparing different ecosystems and depths. In coastal upwelling systems (e.g., the U.S. West Coast), the intrusion of low-pH, corrosive water is episodic and strongly coupled with seasonal upwelling events [126]. This creates a “pH-pulse” environment where mesozooplankton experience intermittent acidification stress, leading to a more dynamic and less predictable cascade disruption compared to the open ocean [127]. By contrast, in polar regions, cold waters naturally have a higher capacity to absorb CO_2_, making them more susceptible to severe and chronic acidification. In the Arctic Ocean, rapidly melting sea ice exacerbates OA, and its impact on the sensory abilities of key species, such as *Calanus*, could fundamentally alter the strong cascades characteristic of these high-latitude systems [92,128]. Furthermore, vertical pH gradients add another layer of complexity. Diel vertical migrators, such as many copepods, are exposed to more severe acidification during their daytime residence in deep, high-CO_2_ waters [100,127]

### 4.3. Eutrophication and Hypoxia

Coastal eutrophication, driven by riverine nutrient input, alters phytoplankton community structure and promotes harmful algal blooms (HABs), disrupting MMTC. Eutrophication stimulates large phytoplankton blooms (e.g., diatoms), increasing direct mesozooplankton grazing and reducing predation on microzooplankton, weakening indirect cascade effects [20]. HAB proliferation further disrupts cascades—toxic phytoplankton species reduce mesozooplankton feeding rates and survival, while non-toxic HAB taxa outcompete smaller phytoplankton, altering prey availability for microzooplankton [20,76]. In the East China Sea estuary, eutrophication-induced HABs reduced mesozooplankton biomass by 25%, leading to increased microzooplankton grazing and a 30% reduction in CS values [76].

Hypoxia, a common consequence of eutrophication, impairs mesozooplankton motility, feeding efficiency, and predator-prey interactions. Low oxygen levels (<2 mg O_2_ L^−1^) reduce mesozooplankton swimming speed and sensory acuity, decreasing predation on microzooplankton by 20–30% [76]. Hypoxic zones also serve as refuges for microzooplankton, increasing their biomass and thereby increasing grazing pressure on phytoplankton, further weakening the cascade effects. In hypoxic hotspots of the northern South China Sea, MMTC intensity was 18–22% lower than in adjacent oxygenated waters, with cascades often shifting from positive to neutral or inverse [76,78].

The combined effects of eutrophication and hypoxia represent a significant threat to the stability and efficiency of marine trophic cascades. The disruption of predator-prey interactions at the base of the food web, coupled with altered energy transfer dynamics, can have far-reaching consequences for higher trophic levels, including fisheries and overall ecosystem health [76].

### 4.4. Multi-Stressor Interactive Effects

As mentioned above, global change stressors rarely act independently, and their interactive effects, whether synergistic, antagonistic, or additive, pose significant challenges to the stability of mesozooplankton-mediated (MMTC) [10,20,76,129]. Single–stressor studies often fail to capture the complex realities of marine ecosystems, especially in coastal and estuarine systems, where local anthropogenic disturbances, such as eutrophication and hypoxia, intersect with global drivers, such as warming and ocean acidification. Synergistic interactions, in which combined stressors exert stronger impacts than the sum of their individual effects, are particularly prevalent in coastal and estuarine systems, where local anthropogenic disturbances (eutrophication, hypoxia) overlap with global drivers (warming, OA) [129].

For example, in the East China Sea estuary, combined warming (2 °C), OA (pH 7.6), and hypoxia (<2 mg O_2_ L^−1^) reduced MMTC intensity by 55%, a far greater reduction than the 15–25% observed for every single stressor [10,76]. This synergy arises from cascading physiological and behavioral impairments: warming increases metabolic demand, OA disrupts sensory cues, and hypoxia limits motility, collectively crippling mesozooplankton predation on microzooplankton.

The context dependency of multi-stressor interactions further complicates predictions. In polar regions, where large-bodied mesozooplankton dominate, combined warming and OA reduce CS values by 30%, whereas in tropical systems with small-bodied taxa, the same stressor combination reduces cascade intensity by 45% [10]. This variability is driven by trait-based differences in thermal tolerance and acidification sensitivity among different mesozooplankton communities, underscoring the critical need for region-specific assessment frameworks. Additionally, temporal variability in stressor exposure—such as seasonal hypoxia—can alter interaction outcomes, with short-term stress pulses often triggering more extreme cascade disruptions than chronic exposure [20].

## 5. Future Research Directions

### 5.1. Integrating Trait-Based Ecology and Mechanistic Modeling

Current MMTC research largely relies on biomass-based metrics, which often fail to fully capture the intricate role of functional traits in modulating cascade dynamics. Future studies should prioritize trait-based frameworks that quantify how morphological (body size, feeding appendage structure), physiological (metabolic rate, assimilation efficiency), and behavioral (prey selection, DVM) traits modulate cascade strength across environmental gradients [130,131]. Integrating these traits into the NPMZ model will enhance predictive accuracy, particularly under global change—for example, incorporating temperature-dependent trait plasticity can improve forecasts of how warming alters mesozooplankton predation on microzooplankton [132]. The strength of trophic cascades, for instance, is more strongly determined by energy transfer efficiency than by primary productivity [133]. Furthermore, coupling trait-based models with biogeochemical models will clarify how MMTC disruption affects carbon sequestration and nutrient cycling, providing critical insights for climate change mitigation strategies [130,132]. For example, the comprehensive field study utilizing compound-specific isotope analysis (CSIA) demonstrates how environmental regulation influences planktonic food web structure and energy transfer efficiency [134]. This type of detailed analysis of trophic pathways is essential for understanding how mesozooplankton and fish larvae are supported in nutrient-poor waters like the Kuroshio [135].

Advances in high-throughput trait measurement technologies are crucial for this shift. Automated image analysis and metabolomics are enabling the generation of comprehensive trait datasets across global marine ecosystems [131]. These datasets can be used to parameterize trait-based models that explicitly link individual-level traits to ecosystem-level cascade effects, addressing the scalability gap between laboratory experiments and open-ocean dynamics [131]. The harmonization of zooplankton trait data from various repositories is already underway to facilitate such comprehensive analyses [131].

### 5.2. Unraveling Cryptic Trophic Interactions

Although literature has revolutionized our understanding of mesozooplankton diet composition, significant gaps remain in quantifying ingestion rates, prey assimilation, and the role of cryptic interactions (e.g., mixotrophy, intraguild predation) [136,137]. Molecular techniques, lipid analysis, and stable isotope analyses, especially compound-specific nitrogen stable isotope analysis of amino acids, are powerful tools for quantifying trophic structure and energy flow within complex food webs [38,137,138,139]. Future research should combine metabarcoding with stable isotope analysis and fatty acid profiling to quantify trophic transfer efficiency and nutritional constraints on MMTC.

The role of mixotrophic protists in MMTC also requires further investigation. These organisms blur traditional trophic boundaries, acting as both producers and consumers, and their responses to global change may reshape cascade pathways [10,137]. Experimental studies examining how warming, OA, and hypoxia alter mixotroph feeding behavior will clarify their contributions to cascade stability [130]. Additionally, intraguild predation among mesozooplankton taxa (e.g., copepods preying on cladocerans) may modulate overall predation pressure on microzooplankton, yet this interaction is rarely incorporated into cascade models [140].

### 5.3. Multi–Stressor Experiments Across Temporal and Spatial Scales

Multi-stressor experiments across nested spatial and temporal scales are crucial for understanding the cumulative impacts of anthropogenic pressures on mesozooplankton trophic cascades. Most existing multi–stressor studies are short-term (days to weeks) and conducted in mesocosms [141], limiting our ability to predict long–term (decadal) cascade responses and scale results to open-ocean ecosystems [142]. Understanding these interactions requires experiments that cover diel to decadal timescales and scales from mesocosms to coastal ecosystems and ocean basins, considering acclimation and evolutionary responses. Eutrophication-driven changes in plankton trophic interactions, influenced by trade-offs in functional traits like growth rate and antipredation defense, further underscore the need for multi–stressor research [143].

Additionally, comparative studies across contrasting marine realms (from eutrophic estuaries to oligotrophic gyres, and polar to tropical systems)are essential to identify generalizable principles governing MMTC responses to multi-stressor environments [12]. Each of these environments presents unique sets of stressors and trophic structures, and by comparing their MMTC responses, researchers can discern universal ecological mechanisms and context-specific adaptations. For example, the trophic ecology of midwater zooplankton has been observed to vary along productivity gradients, demonstrating the influence of environmental conditions on food web structure and energy transfer efficiency [134]. Similarly, studies in areas such as the Kuroshio and its neighboring waters have highlighted how mesozooplankton and fish larvae are sustained even in areas with low phytoplankton standing stocks, emphasizing the need to understand diverse trophic pathways across different marine biomes [135]. Integrating these data into global synthesis analyses will enable the development of predictive frameworks that account for regional variability [12]. Machine-learning algorithms, for instance, are increasingly being employed to model global mesozooplankton biomass and identify relevant controlling mechanisms, providing a path toward more sophisticated predictive capabilities [132].

### 5.4. Advancing Technological and Methodological Innovations

Advancing technological and methodological innovations will significantly enhance the ability to study mesozooplankton. AI–powered imaging technologies, such as ZooScan and EcoTaxa, alongside environmental DNA/RNA metabarcoding, offer high-throughput, taxonomically resolved identification of species and their interactions [144].

Autonomous underwater vehicles (AUVs) equipped with high-resolution imaging and sampling tools can provide spatiotemporal data on plankton communities at scales previously unattainable, reducing sampling bias and improving our understanding of cascade heterogeneity [135]. Similarly, in situ sensor networks can monitor environmental stressors and cascade-related metrics in real time (e.g., mesozooplankton biomass, phytoplankton size structure), enabling early detection of cascade disruption [12].

Methodological innovations in grazing rate estimation are also poised to enhance the accuracy of cascade strength quantification. The integration of advanced techniques, such as the three-level correction model with in situ imaging, can address historical underestimation of grazing rates [12,14]. Furthermore, machine-learning algorithms can analyze large datasets from AUVs and sensors to identify hidden patterns in MMTC dynamics, such as threshold effects where stressor exposure triggers abrupt cascade shifts. These innovations will enhance our ability to predict, monitor, and mitigate the impacts of global change on MMTC.

## 6. Conclusions

Mesozooplankton-mediated trophic cascades (MMTC) play a central role in regulating marine ecosystem dynamics and biogeochemical cycles. This review presents an integrated quantitative framework—including a dual-pathway cascade model, a grazing rate correction method, a cascade strength index, and an NPMZ model—to better quantify and predict MMTC. These tools help clarify how direct predation and trait-mediated interactions jointly shape cascade outcomes under varying environmental conditions.

MMTC exhibits strong spatiotemporal heterogeneity, influenced by physical processes such as mixed-layer dynamics and ocean currents, as well as biotic feedback loops. Cascade strength varies systematically across regions, from high values in polar systems dominated by large-bodied zooplankton to lower values in tropical oligotrophic waters. Temporal dynamics, including diel vertical migration and seasonal succession, further modulate cascade intensity and ecosystem outcomes.

Global change stressors—particularly warming, acidification, eutrophication, and hypoxia—interact in complex, often synergistic ways to disrupt MMTC. These disruptions alter mesozooplankton feeding behavior, decouple trophic linkages, and can lead to cascade collapse, with implications for carbon export and fisheries sustainability. Looking ahead, ongoing global climate change will continue to reshape MMTC through multifaceted pathways: persistent warming will drive latitudinal shifts in mesozooplankton communities (e.g., expansion of small-bodied tropical taxa into temperate regions), alter thermal optima for key species, and exacerbate trophic mismatches between mesozooplankton reproduction and prey availability; ocean acidification will further impair sensory and behavioral traits critical for selective feeding, especially in polar and coastal upwelling systems with low buffering capacity; and the intensification of eutrophication and hypoxia will disrupt predator-prey interactions in coastal ecosystems, weakening top-down control. Collectively, these changes will modify plankton community size structure, reduce biological carbon pump efficiency, and threaten the stability of marine food webs and the provisioning of ecosystem services such as fisheries support.

Moving forward, research must integrate trait-based ecology, mechanistic modeling, and innovative technologies such as AI-driven imaging and environmental DNA. Cross-system comparisons and multi-stressor experiments across scales are essential to develop predictive frameworks that support ecosystem resilience and informed management in a changing ocean.

## Figures and Tables

**Table 2:** NPMZ model parameter ranges and references.

Parameter	Range	Unit	Reference
$r_{\max}$	0.5–2.0	d^−1^	[27,49]
$k_{N}$	0.1–1.0	μmol N L^−1^	[14,49]
$g_{M}$	0.3–1.5	d^−1^	[14,16,27]
$G_{Z}$	0.2–1.2	d^−1^	[14,16]
$\gamma_{M},\;\gamma_{Z}$	0.2–0.4	Dimensionless	[38,52]
$l_{\mathrm{PN}},{\;l}_{\mathrm{MN}},\;l_{\mathrm{ZN}}$	0.05–0.2	d^−1^	[14,16]

## Data Availability

No new data were created or analyzed in this study. Data sharing is not applicable to this article.
